# Effects of two consecutive mixed meals high in palmitic acid or stearic acid on 8-h postprandial lipemia and glycemia in healthy-weight and overweight men and postmenopausal women: a randomized controlled trial

**DOI:** 10.1007/s00394-021-02530-2

**Published:** 2021-03-17

**Authors:** Merel A. van Rooijen, Jogchum Plat, Peter L. Zock, Wendy A. M. Blom, Ronald P. Mensink

**Affiliations:** 1 Department of Nutrition and Movement Sciences, NUTRIM (School of Nutrition and Translational Research in Metabolism), Maastricht University Medical Center, Maastricht, The Netherlands; 2 Unilever Research and Development, Wageningen, The Netherlands

**Keywords:** Stearic acid, Palmitic acid, Human intervention study, Postprandial, Lipemia, Glycemia

## Abstract

**Purpose:**

Palmitic and stearic acids have different effects on fasting serum lipoproteins. However, the effects on postprandial lipemia and glycemia are less clear. Also, the effects of a second meal may differ from those of the first meal. Therefore, we studied the effects of two consecutive mixed meals high in palmitic acid- or stearic acid-rich fat blends on postprandial lipemia and glycemia.

**Methods:**

In a randomized, crossover study, 32 participants followed 4-week diets rich in palmitic or stearic acids, At the end of each dietary period, participants consumed two consecutive meals each containing ± 50 g of the corresponding fat blend.

**Results:**

Postprandial concentrations of triacylglycerol (diet-effect: − 0.18 mmol/L; *p* = 0.001) and apolipoprotein B48 (diet-effect: − 0.68 mg/L; *p* = 0.002) were lower after stearic-acid than after palmitic-acid intake. Consequently, total (iAUC_0–8 h_) and first meal (iAUC_0–4 h_) responses were lower after stearic-acid intake (*p* ≤ 0.01). Second meal responses (iAUC_4–8 h_) were not different. Postprandial changes between the diets in non-esterified fatty acids (NEFA) and C-peptide differed significantly over time (*p* < 0.001 and *p* = 0.020 for diet*time effects, respectively), while those for glucose and insulin did not. The dAUC_0–8 h_, dAUC_0–4 h_, and dAUC_4–8 h_ for NEFA were larger after stearic-acid intake (*p* ≤ 0.05). No differences were observed in the iAUCs of C-peptide, glucose, and insulin. However, second meal responses for glucose and insulin (iAUC_4–8 h)_ tended to be lower after stearic-acid intake (*p* < 0.10).

**Conclusion:**

Consumption of the stearic acid-rich meals lowered postprandial lipemia as compared with palmitic acid. After the second stearic acid-rich meal, concentrations of C-peptide peaked earlier and those of NEFA decreased more.

*Clinical trial registry* This trial was registered at clinicaltrials.gov as NCT02835651 on July 18, 2016.

**Supplementary Information:**

The online version contains supplementary material available at 10.1007/s00394-021-02530-2.

## Introduction

As we spend most of the day in a postprandial state, understanding relations between diet-induced postprandial physiological changes and cardiometabolic health is important. Indeed, it has been shown that elevated and prolonged postprandial lipemia and glycemia are associated with an increased risk to develop cardiovascular disease (CVD) [1, 2]. However, for dietary fat intake, recommendations are mainly based on effects on fasting serum LDL-cholesterol (LDL-C) concentrations, an established CVD-risk factor [3]. It is for example well-known that replacing saturated fatty acids with unsaturated fatty acids has a beneficial effect on LDL-C [4]. Saturated fat, however, is an umbrella term for different saturated fatty acids that exert different metabolic effects. Of these, palmitic acid (C16:0) and stearic acid (C18:0) are the most commonly consumed saturated fatty acids in the Western diet. It is well established that palmitic acid increases fasting serum LDL-C concentrations compared with stearic acid. However, the effects of these saturated fatty acids on postprandial metabolism are less clear. Attenuated postprandial lipemia after acute intake of stearic acid compared with palmitic acid has been observed in two studies [5, 6], but not in other studies [7–11]. One hypothesis is that stearic acid-rich fats delay fat digestion and absorption, because of the presence of more fat solids at body temperature due to its higher melting range [12]. So far, no differences between palmitic-acid and stearic-acid intake have been found in postprandial responses of glucose [6, 13] and insulin [5, 8, 13].

In daily-life, people generally consume multiple meals a day and lipids ingested during the first meal will also appear in the circulation when a second meal is consumed, even if this second meal is low in fat [14, 15]. Previous studies examining acute effects of palmitic-acid versus stearic-acid intakes provided a low-fat lunch after 3 to 4 h [6–9]. However, as most meals during the day provide fats, it is of interest to examine if postprandial responses of consecutive meals high in fat differ from those after a single meal. Therefore, we have examined the effects of two consecutive mixed meals high in palmitic acid- or stearic acid-rich fat blends on postprandial lipemia and glycemia during an 8-h period. Postprandial tests were performed after subjects had consumed 4-week diets rich in the corresponding fatty acid.

## Methods

This postprandial intervention study was part of a double-blind, randomized, crossover study that consisted of two 4-week intervention periods during which healthy-weight or overweight men and women received products enriched with either palmitic acids (C16:0) or stearic acids (C18:0). Effects of the 4-week diets on fasting cardiometabolic risk markers and details of the study design have been described previously [16]. Compared with the diet rich in palmitic acid, intake of palmitic acid was 6.0 percent of energy (En%) lower when subjects consumed the diet rich in the stearic acid diet (*p* < 0.001) and intake of stearic acid 6.5 En% higher. Oleic-acid intake was slightly higher on the stearic-acid diet (+ 0.4 En%) and fiber intake was − 1.3 g lower. Intakes of energy, other macronutrients, and cholesterol were comparable between the two intervention periods, which were separated by a wash-out period of at least 4 weeks (Supplemental table 1). At the end of each intervention period, an 8-h postprandial test was performed for which participants consumed a mixed meal high in either palmitic acid- or stearic acid-rich fat blends. Participants received a second meal 4 h after the first meal to induce a second-meal effect.

### Participants

Briefly, healthy men and women were recruited from Maastricht and surrounding areas and met the following criteria: aged between 45 and 70 years, postmenopausal (women), BMI between 18 and 30 kg/m^2^ with a stable body weight during the last 3 months (< 3 kg change), no cardiovascular disease or medical condition that might interfere with the study. Participants were included if they were healthy, which was based on a medical questionnaire, had fasted serum total cholesterol (TC) concentrations < 8.0 mmol/L and triacylglycerol (TAG) concentrations < 4.5 mmol/L, and plasma HbA1c concentrations < 48 mmol/mol (or 6.5%). After screening, 41 participants were included. All participants gave their written informed consent before entering the study. The Medical Ethical Committee of the MUMC + had approved the protocol. The study was registered at ClinicalTrials.gov with identifier NCT02835651.

### Study design and meals

For the postprandial test at the end of each intervention period, participants were asked to refrain from strenuous exercise 48 h before this test day. After measuring weight, blood pressure, and obtaining a fasted blood sample via venepuncture, an intravenous cannula was placed in an antecubital vein and another fasting blood sample was collected (T0). Participants then received a mixed meal provided as a shake, which they were asked to consume within 5 min. This meal contained 46.6 g of the fat blend that participants also received during the preceding 4-week intervention period. The composition of the meal was similar for all participants and each meal consisted of 50 g of fat, 5 g of protein, and 54 g of carbohydrates (**Table ****1**). This amount of fat was chosen, because it represents a realistic fat load in a Western dinner and causes a clear increase in serum TAG concentrations [17]. 4 h after the first meal, participants consumed a second meal with the same composition as the first one. Postprandial blood samples were taken at 15 (T15), 30 (T30), 45 (T45), 60 (T60), 90 (T90), 120 (T120), 180 (T180), 240 (T240), 300 (T300), 360 (T360), 420 (T420), and 480 (T480) minutes after shake consumption. Immediately after T240 (approximately around lunch time), the second meal was consumed. During the entire test day, participants were not allowed to drink anything—except for water—or to eat. Participants were asked to keep the amount of water consumption comparable between both test days.

**Table 1:** Nutrient composition of the standardized test shakes provided for breakfast and lunch

	Palmitic acid-rich shake	Stearic acid-rich shake
Energy (kcal)	697.4	697.4
Carbohydrates (en%)	30.8	30.8
Protein (en%)	4.8	4.8
Fat (en%)	64.5 (50 g)	64.5 (50 g)
SFA (en%)	33.0	33.0
C16:0 (en%)	28.3 (22 g)	3.1 (2.5 g)
C18:0 (en%)	3.2 (2.4 g)	29.2 (22.6 g)
MUFA (en%)	26.0	26.4
C18:1 (en%)	25.7	26.1
PUFA (en%)	4.7	4.7
C18:3 n-3 (en%)	0.1	0.4
Cholesterol (mg)	120	120
Fiber (g)	1.28	1.28

*SFA*, saturated fatty acids; *MUFA*, cis-monounsaturated fatty acids; *PUFA*, cis-polyunsaturated fatty acids

### Experimental fat blends

Both blends of natural fats were provided by Unilever R&D (Vlaardingen, Netherlands). For the palmitic acid-rich blend, a mix of 90% palm oil mid-fraction (POM) and 10% high oleic sunflower oil (HOSO) was used. For the stearic acid-rich blend, a mix of 92% allanblackia oil (AB) and 8% sunflower oil (SO) was used. Fat blends were comparable in saturated, monounsaturated and polyunsaturated fatty acid content (Supplemental table 2). Slip melting points for the palmitic acid- and stearic acid-rich fat blend were respectively 33.9 °C and 40.5 °C, and the solid fat contents at 37 °C were 1 and 8%.

### Blood collection and biochemical analyses

Blood was sampled in serum separator vacutainer tubes (Becton, Dickinson and company, NJ, USA) for analyses of triacylglycerol (TAG), apolipoprotein B48 (apoB48), non-esterified fatty acids (NEFA), insulin, and C-peptide. After sampling, serum tubes were allowed to clot for at least 30 min at room temperature and subsequently centrifuged at 1300×*g* for 15 min at 20 °C. Blood for glucose analysis was sampled in NaF-plasma vacutainer tubes (Becton, Dickinson and company) and directly put on ice after sampling with subsequent centrifugation at 1300×*g* for 15 min at 4 °C. Aliquots of serum and plasma samples were snap-frozen in liquid nitrogen and stored at − 80 °C until analysis.

Serum concentrations of TAG corrected for free glycerol (GPO Trinder; Sigma-Aldrich, Missouri, USA) and apoB48 (ELISA; Shibayagi Co., Shibukawa Japan) were measured at all timepoints except for T15 and T45. Concentrations of serum NEFA (Wako Chemicals GmbH, Neuss, Germany), serum insulin and C-peptide (Linco Research, Missouri, USA), and plasma glucose (Horiba ABX) were measured at all time points. Samples from one subject were analyzed within the same analytical run.

### Statistical analyses

The primary outcome parameter of this study was the change in fasting cholesterol efflux capacity for which a power calculation was performed (4). Here we report the results on other outcome parameters. Data are reported as least squared mean (LSM) with 95% confidence interval (CI) unless otherwise indicated. Postprandial time curves were analyzed using linear mixed models with participants as between subject variable, baseline values of the corresponding day (T0) as the covariate, and period, diet, time, diet*time, and baseline as fixed factors. If the diet*time interaction term did not reach statistical significance, indicating that responses were similar at all time points, it was omitted from the model. In this model, the statistical significance of the factor diet indicated that differences between the stearic acid and palmitic acid diets were similar at all time points. Differences are reported as least squared means (LSM) with 95% confidence interval (CI). Sex effects were also determined by addition of sex, diet*sex, time*sex, and diet*sex*time as fixed factors to the model. However, for none of the parameters sex effects were present and therefore omitted from the model. Incremental areas under the curve (iAUC) or decremental areas under the curve (dAUC) were calculated for all parameters using the trapezoidal rule as previously described [18]. We assessed the total postprandial response (0 to 8 h; i/dAUC_0–8 h_), as well as the first meal response (0 to 4 h; i/dAUC_0–4 h_) and the second meal response (4 to 8 h; i/dAUC_4–8 h_). Peak increases or decreases were calculated by comparing maximal changes during the 8 h postprandial follow-up to T0 (max_0–8 h_). Maximal changes after the first meal were calculated by comparing concentrations between T0 and T240 to the concentrations at T0 (max_0–4 h_) and maximal changes after the second meal were calculated by comparing concentrations between T240 and T480 to the concentrations at T240 (max_4–8 h_). i/dAUC differences and maximal increases were assessed using linear mixed models with subject as a random factor, and period and diet as fixed factors, and reported as LSM with 95% CI. Sex effects were also tested for i/dAUCs by addition of sex and diet*sex to the model as fixed factors but the diet*sex interaction term was not significant for any of the parameters and thus omitted. Results were considered statistically significant if *p* < 0.05. All analyses were performed using IBM SPSS Statistics for Mac, version 24.0 (IBM Corp. Armonk, NY, USA). Blinding was maintained until all analyses were performed.

## Results

The participant flow throughout the study is shown in Supplemental fig. S1. Fifty-eight participants were assessed for eligibility of which 41 were included and randomly allocated to the intervention periods. Seven participants withdrew during the first week of the first intervention period as described previously [16]. Of the remaining 34 participants, one man and one woman did not complete one or both postprandial test day(s) due to nausea. In the end, 32 participants (19 men and 13 women) completed both postprandial test days and were included in the analyses. Of these, 10 men and 7 women started with the palmitic-acid diet, and 9 men and 6 women with the stearic-acid diet. Characteristics of the participants at screening are shown in Supplemental Table 3.

### Postprandial lipemia

**Fig. 1:**
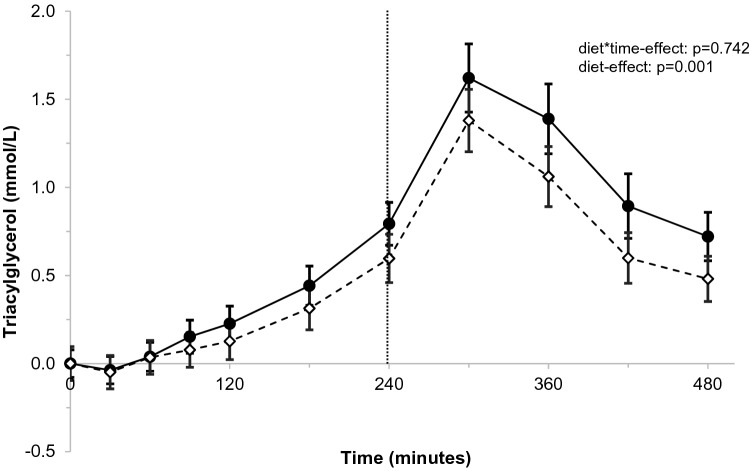
Postprandial changes in triacylglycerols (TAG; mmol/L) over time after meals rich in palmitic acid (•) or stearic acid (⋄)^a^. ^a^TAG concentrations were measured at baseline, and 30, 60, 90, 120, 180, 240, 300, 360, and 480 min after meal intake. After 240 min, a second meal was consumed that was similar to the first meal. Postprandial time curves were analyzed using linear mixed models. *N* = 32. A significant overall diet-effect was observed (*p* = 0.001)

For postprandial TAG concentrations, there were no significant differences between the meals rich in palmitic acid or stearic acid at the various time points of the postprandial response (*p* = 0.742 for diet*time interaction; Fig. 1). However, a significant diet-effect was observed, i.e. postprandial TAG concentrations after stearic-acid intake were on average 0.18 mmol/L lower (*p* = 0.001) over the total 8-h follow-up period. Consequently, the iAUC_0–8 h_ (*p* = 0.002) and peak values (TAG_max0–8 h_; p = 0.003) were lower after stearic-acid intake (Supplemental table 4). During the 4 h after the first meal, comparable results were observed as over the whole 8-h follow-up period, i.e. the iAUC_0–4 h_ and TAG_max0–4 h_ were lower (p = 0.007 for both) after the stearic-acid meal. The iAUC_4–8 h_ in the last 4 h after the second meal was not statistically different between palmitic acid and stearic acid (*p* = 0.127), but peak values tended to be lower after stearic-acid intake (TAG_max4–8 h_; *p* = 0.079).

**Fig. 2:**
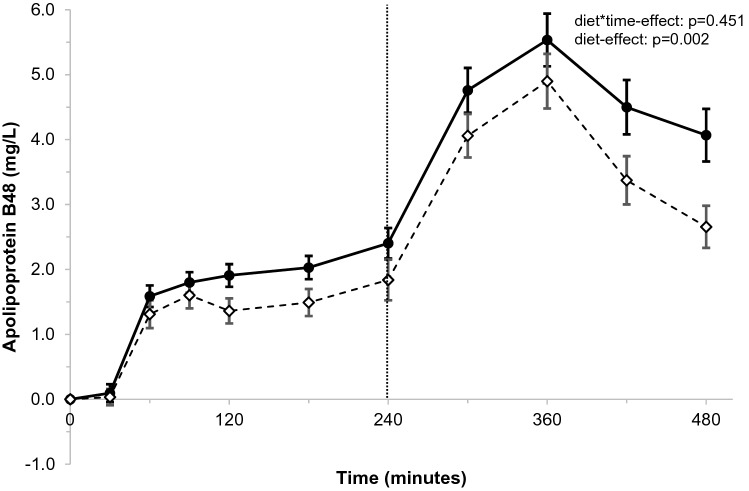
Postprandial changes in apolipoprotein B48 (apoB48; mg/L) over time after meals rich in palmitic acid (•) or stearic acid (⋄)^a^. ^a^ApoB48 concentrations were measured at baseline, and 30, 60, 90, 120, 180, 240, 300, 360, and 480 min after meal intake. After 240 min, a second meal was consumed that was similar to the first meal. Postprandial time curves were analyzed using linear mixed models. *N* = 32. A significant overall diet-effect was observed (*p* = 0.002)

Differences in postprandial changes at the various timepoints for apoB48 were comparable (*p* = 0.451 for diet*time interaction; Fig. 2). Like for TAG, average apoB48 concentrations were overall lower after stearic-acid than after palmitic-acid intake (diet-effect: − 0.68 mg/L; *p* = 0.002), as also shown by the lower iAUC_0–8 h_ (*p* = 0.008) and peak values (ApoB48_max0–8 h_; *p* = 0.034; Supplemental table 5). After the first meal, also a lower iAUC_0–4 h_ (*p* = 0.010) and ApoB48_max0–4 h_ (*p* = 0.048) were observed after intake of stearic acid. After the second meal, the iAUC_4–8 h_ (p = 0.355) and ApoB48_max4–8 h_ (*p* = 0.585) were not different between palmitic-acid and stearic-acid intakes. Subsequently, postprandial changes in TAG:apoB48 ratio at the various timepoints were comparable between palmitic-acid and stearic-acid intakes (*p* = 0.237 for diet*time interaction; Supplemental Fig. 2) and no diet-effects were observed (*p* = 0.881).

### Postprandial glycemia

**Fig. 3:**
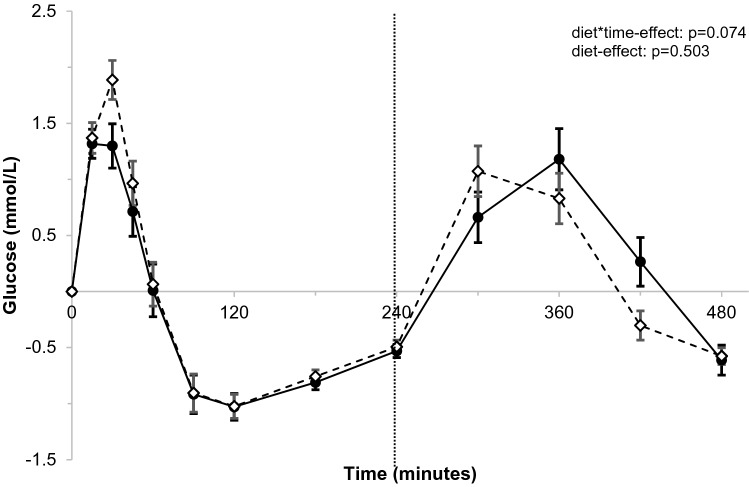
Postprandial changes in glucose (mmol/L) over time after meals rich in palmitic acid (•) or stearic acid (⋄)^a^. ^a^Glucose concentrations were measured at baseline, and 15, 30, 45, 60, 90, 120, 180, 240, 300, 360, and 480 min after meal intake. After 240 min, a second meal was consumed that was similar to the first meal. Postprandial time curves were analyzed using linear mixed models. *N* = 32

For postprandial glucose concentrations, postprandial responses after palmitic-acid and stearic-acid intakes at the various time points did not differ significantly (*p* = 0.074 for diet*time interaction; Fig. 3). Also, no diet-effects were observed (*p* = 0.503). The iAUC_0–8 h_ (*p* = 0.375) and peak values (Glucose_max0–8 h_; *p* = 0.876) were comparable between palmitic-acid and stearic-acid intakes (Supplemental table 6). After the first meal, the iAUC_0–4 h_ (*p* = 0.362) was also not different, but the Glucose_max0–4 h_ tended to be higher after stearic-acid intake (*p* = 0.059). In contrast to the 4 h after the first meal, the iAUC_4–8 h_ (*p* = 0.095) and the Glucose_max4–8 h_ (*p* = 0.064) after the second meal tended to be lower after stearic-acid intake.

**Fig. 4:**
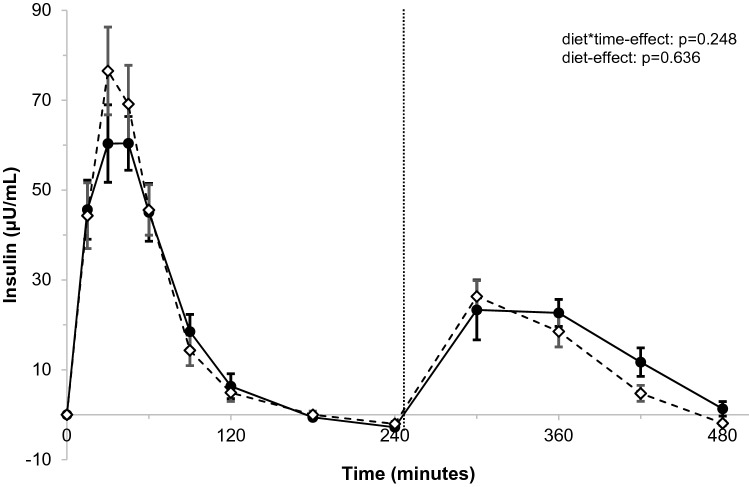
Postprandial changes in insulin (μU/mL) over time after meals rich in palmitic acid (•) or stearic acid (⋄)^a^. ^a^Insulin concentrations were measured at baseline, and 15, 30, 45, 60, 90, 120, 180, 240, 300, 360, and 480 min after meal intake. After 240 min, a second meal was consumed that was similar to the first meal. Postprandial time curves were analyzed using linear mixed models. *N* = 32

Differences in postprandial changes at the various timepoints in insulin concentrations were comparable between the palmitic-acid and stearic-acid meals (*p* = 0.248 for diet*time interaction; Fig. 4), and also no diet-effects were observed (*p* = 0.636). The iAUC_0–8 h_ (*p* = 0.404) and Insulin_max0–8 h_ (*p* = 0.483) did not differ between palmitic-acid and stearic-acid intakes, and similar results were observed during the 4 h after the first meal (Supplemental table 7). After the second meal, the insulin iAUC_4–8 h_ tended to be lower after intake of stearic acid (*p* = 0.064), while insulin_max4–8 h_ was not different (*p* = 0.115).

**Fig. 5:**
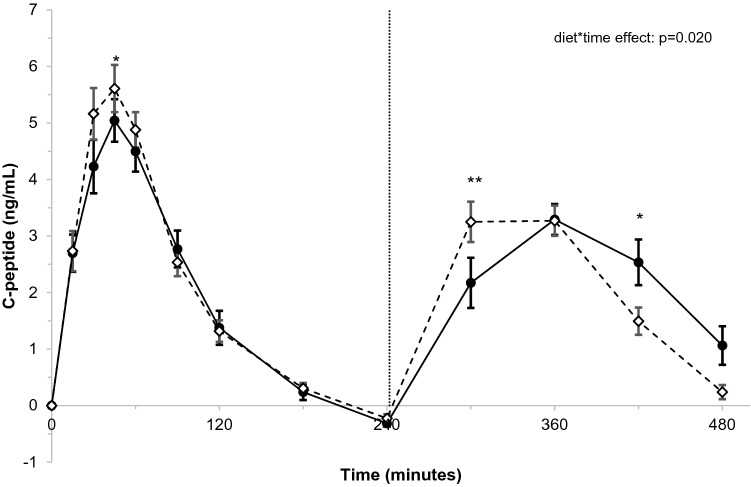
Postprandial changes in C-peptide (ng/mL) over time after meals rich in palmitic acid (•) or stearic acid (⋄)^a^. ^a^C-peptide concentrations were measured at baseline, and 15, 30, 45, 60, 90, 120, 180, 240, 300, 360, and 480 min after meal intake. After 240 min, a second meal was consumed that was similar to the first meal. Postprandial time curves were analyzed using linear mixed models. *N* = 32. A significant diet*time interaction was observed (*p* = 0.020)

Postprandial changes in C-peptide concentrations at the various timepoints differed between palmitic acid and stearic acid (*p* = 0.020 for diet*time interaction; Fig. 5). Compared with palmitic acid, stearic-acid intake resulted in significantly higher C-peptide concentrations 30 and 300 min postprandially (+ 1.01 ng/mL; *p* = 0.011 and + 1.16 ng/mL; *p* = 0.004 respectively), but in lower concentrations at 420 min (− 0.96 ng/mL; *p* = 0.015). No differences between palmitic-acid and stearic-acid intakes were found in iAUCs and peak values over the total 8-h follow up, or in those over the 4 h follow up after the first and second meals (Supplemental table 8).

**Fig. 6:**
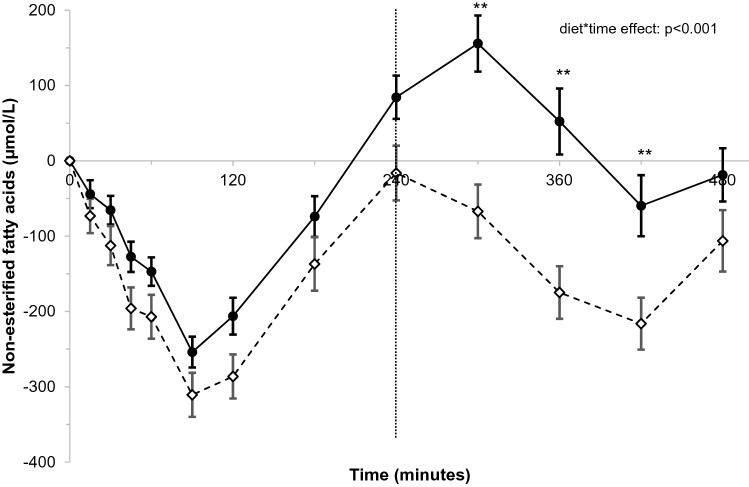
Postprandial changes in non-esterified fatty acids (NEFA; μmol/L) over time after meals rich in palmitic acid (•) or stearic acid (⋄)^a^. ^a^NEFA concentrations were measured at baseline, and 15, 30, 45, 60, 90, 120, 180, 240, 300, 360, and 480 min after meal intake. After 240 min, a second meal was consumed that was similar to the first meal.. Postprandial time curves were analyzed using linear mixed models. *N* = 32. A significant diet*time interaction was observed (*p* < 0.001)

The postprandial time curves of NEFAs were different between the palmitic-acid and stearic-acid meals (*p* < 0.001 for diet*time interaction; Fig. 6). NEFA concentrations were lower after the stearic-acid meals at T300 (− 177 μmol/L; *p* < 0.001), T360 (− 181 μmol/L; *p* < 0.001), and T420 (− 111 μmol/L; *p* = 0.001). The postprandial dAUC_0–8 h_ (*p* = 0.005) and maximal decrease of NEFA (NEFA_max0–8 h;_ *p* = 0.026) were more pronounced after stearic-acid intake (Supplemental table 9). Similarly, in the 4 h after the first meals, the dAUC_0–4 h_ was more pronounced after intake of stearic acid (*p* = 0.025) and the maximal decreases tended to be more pronounced (NEFA_max0–4 h_; *p* = 0.054). After the second meal, the dAUC_4–8_ also tended to be more pronounced after stearic-acid intake (*p* = 0.054), but the maximal decreases did not differ (NEFA_max4–8 h_; *p* = 0.499).

## Discussion

Results of this double-blind randomized study indicate that fats rich in palmitic acid or stearic acid, the two major saturated fatty acids in most Western diets, differently affect postprandial metabolism. Effects were studied after intakes of two consecutive mixed meals high in palmitic acid- or stearic acid-rich fat blends and tests were performed after subjects had consumed for 4 weeks diets rich in the corresponding fatty acid.

### Postprandial lipemia

Postprandial lipemia was decreased after intake of the stearic acid-rich meals compared with the palmitic acid-rich meals, as indicated by lower TAG and apoB48 concentrations. Differences between the meals, as indicated by the iAUCs, were in particular evident after the first meal. Earlier studies on postprandial lipid responses between palmitic acid and stearic acid-rich meals were not consistent. In most studies [7–11], no clear differences were observed, although in one study lower TAG concentrations were reported 3 h after intake of the meal rich in stearic acid [9]. In two other studies, however, lower TAG concentrations were observed after intake of a stearic acid-rich meal (lard) as compared with a palmitic acid-rich meal (palm olein) [5, 6]. These lower TAG concentrations—which agree with our findings—may relate to the physical characteristics of the fat sources used, especially to those of stearic acid, and not by the fatty-acid composition per se. It has been suggested that postprandial lipemia is attenuated if the fat is not fully liquid at 37 °C [6, 19]. Indeed, lard had a higher percentage of solids at 37 °C than palm olein [6] and in our study the stearic-acid fat blend also had more solids at 37 °C than the palmitic-acid blend. We also observed a lower apoB48 response after intake of the stearic-acid meals. As each chylomicron particle carries one apoB48, this indicates that the number of chylomicrons after stearic-acid intake was lower. In only one other study, apoB48 responses were measured and concentrations tended to be lower after lard intake than after palm-olein intake [6]. This is in line with the hypothesis that a higher solid fat content at 37 °C decreases or delays the absorption rate, resulting in less formation of chylomicron particles and consequently attenuated lipemia [19].

Participants consumed a second, identical meal 4 h after intake of the first meal. After this second meal, differences between palmitic acid and stearic acid on postprandial lipemia were less pronounced. As TAG concentrations were still increasing 4 h after the first meal, it can be speculated that not all the fat was absorbed within this time period, thereby increasing variability in responses and masking possible differences between the two saturated fatty acids after the second meal.

Irrespective of the fatty-acid composition of the meals, serum TAG concentrations already peaked 1 h after the second meal and then started to decrease, while TAG concentrations increased for up to 4 h after the first meal. The rapid increase in TAG after the second meal may have been caused by a release of chylomicron particles that were already formed after the first meal and stored within the enterocyte [15]. This phenomenon was also observed by Baumgartner et al. [20]. In contrast, Tushuizen et al. observed a TAG peak 2 h after a second meal [21]. However, in that study blood was sampled at 2-h intervals and the mixed meals were provided as solid foods, while we and Baumgartner et al. [21] sampled every hour and provided the meals as a shake, which may have increased gastric emptying. Remarkably, the apoB48 peak after the second meal occurred 1 h later than the TAG peak, while after the first meal, both apoB48 and TAG concentrations increased continuously. This was also observed by Baumgartner et al. [20] especially in participants aged between 53 and 69 years of age, which is comparable to the age of our study population. The mechanism underlying this delayed apoB48 peak compared with the TAG peak remains to be determined, but it is possible that during the first hour after the second meal, larger TAG-rich chylomicrons are secreted, as suggested by the higher TAG:apoB48 ratio at 300 min postprandially compared to 60 min. Alternatively, it can be speculated that the contribution of VLDL-TAG to total TAG in the circulation is larger in the first hour after the second meal.

### Postprandial glycemia

C-peptide concentrations were higher after the first stearic acid-rich meal and peaked earlier after the second stearic-acid rich meal as compared with corresponding palmitic-acid meals. Postprandial insulin responses were however not different between palmitic-acid and stearic-acid intakes. For glucose, a comparable pattern as for C-peptide was observed, although these differences did not reach statistical significance. After both meals, postprandial NEFA suppression was more pronounced after stearic-acid intake, in particular after the second meal. Earlier studies that used a single fat-rich meal challenge did not observe any differences between palm olein or palm oil and lard on glucose [5, 13], insulin [5, 8, 13], or C-peptide [13] responses. In two studies, postprandial NEFA concentrations were lower after lard than after palm olein intake [5, 6]. Interesterified palm olein, however, had the same effect as lard, indicating that the observed differences in NEFA were most likely due to the physical characteristics of the fats rather than the fatty-acid compositions [6]. Circulating NEFAs are the resultant of adipocyte lipolysis, NEFA spillover from hydrolysis of circulating TAG-rich lipoproteins, and NEFA uptake and re-esterification [22]. We can only speculate which of these processes was mostly affected by intakes of the palmitic or stearic acid-rich fat blends. Linked to the lower TAG concentrations, the larger decrease in NEFA after stearic-acid intake may be caused by less spillover of NEFAs after hydrolysis of TAG-rich lipoproteins. In addition, increased or earlier insulin secretion after the stearic-acid meals as suggested by differences in C-peptide concentrations may have played a role.

In contrast to postprandial lipemia, differences between palmitic-acid and stearic-acid intakes on parameters related to postprandial glycemia were most pronounced after the second meal. This so-called second-meal effect emphasizes the importance of including second meal challenges to understand dietary effects on postprandial metabolism.

### Limitations and conclusions

In the present study, blood was sampled at 15 min-intervals after the first meals and at 1 h-intervals after the second meals. We can therefore not exclude that the true peaks of glucose, insulin, and C-peptide after the second meals were missed. Also, palmitic acid- or stearic acid-rich meal challenges were performed after 4-week diets enriched with the corresponding fatty acid. Although this is certainly a strength as results more mimic the real life situation, it is not known to what extent our results can be compared to acute studies.

In conclusion, our data demonstrate that the fat blend rich in stearic acid lowered postprandial lipemia as compared with the fat blend rich in palmitic acid. These effects were most pronounced after intake of the first meal. Differences in C-peptide and NEFA concentrations were more evident after intake of the second meal, i.e. intake of the stearic acid-rich fat resulted in an earlier peak of C-peptide concentrations and more pronounced decrease in NEFA concentrations. Translation of these findings into health effects on the long-term needs further study.

## Supplementary Information

Below is the link to the electronic supplementary material.

Supplementary file 1 (PDF 306 KB)

## Data Availability

Not applicable.
