# Supplementary material for: Effects of two consecutive mixed meals high in palmitic acid or stearic acid on 8-h postprandial lipemia and glycemia in healthy-weight and overweight men and postmenopausal women: a randomized controlled trial
Source: Eur J Nutr. 2021 Mar 17;60(7):3659–67. doi: 10.1007/s00394-021-02530-2 (PMC8437914; doi:10.1007/s00394-021-02530-2)
Supplement: Supplementary file 1 — (PDF 306 KB) [file 394_2021_2530_MOESM1_ESM.pdf]

Supplemental materials

Effects of two consecutive mixed meals high in palmitic acid or stearic acid on 8-hour postprandial lipemia and glycemia in healthy-weight and overweight men and postmenopausal women: a randomized controlled trial

Merel A. van Rooijen, Jogchum Plat, Peter L. Zock, Wendy A.M. Blom, Ronald P. Mensink*

*Corresponding author, e-mail address: r.mensink@maastrichtuniversity.nl

Supplemental table 1 Composition of the diets during the intervention periods[a]

	**Palmitic acid**	**Stearic acid**	**P-value[b]**
Energy (kcal)	2448 ± 310	2414 ± 313	0.282
Carbohydrates	46.3 ± 3.8	45.9 ± 3.9	0.464
Protein	15.3 ± 1.3	15.1 ± 1.5	0.388
Fat	36.4 ± 3.3	36.9 ± 3.6	0.250
SFA	15.5 ± 1.7	15.7 ± 1.8	0.257
C16:0	10.2 ± 0.9	4.1 ± 0.5	<0.001
C18:0	2.2 ± 0.3	8.7 ± 0.9	<0.001
Cis-MUFA	14.0 ± 1.5	14.3 ± 1.8	0.167
C18:1	11.3 ± 1.5	11.6 ± 1.8	0.019
Cis-PUFA	5.3 ± 1.0	5.2 ± 1.0	0.332
C18:2 n-6	4.3 ± 0.8	4.2 ± 0.8	0.289
C18:3 n-3	0.64 ± 0.24	0.68 ± 0.23	0.122
Alcohol	2.0 ± 2.1	2.0 ± 2.1	0.420
Cholesterol (mg)	371 ± 49	368 ± 51	0.633
Fibre (g)	29.3 ± 4.4	28.1 ± 3.7	0.041

[a]Values are means ± SD and expressed in % of energy, unless otherwise noted. Values were obtained from Food Frequency Questionnaires.

[b]Differences between the two diets were analyzed using linear mixed models.

Supplemental table 2 Fatty acid composition (w/w) of the experimental fat blends[a]

	**Palmitic acid-rich fat blend**	**Stearic acid-rich fat blend**
Fat blend	0.9 POM/0.1 HOSO	0.92 AB/0.08 SO
SAFA (%)	50.4	50.1
C16:0	43.5	3.2
C18:0	4.6	46.1
MUFA (%)	38.8	39.6
C18:1	38.6	39.4
PUFA (%)	7.2	6.6
C18:3 n-3	0.2	0.6
SMP (°C)	33.9	40.5
Solid fat 37°C (%)	1	8

[a]Percentages of fatty acids are calculated based on total triacylglycerol content (including glycerol). *POM*, Palm oil mid-fraction; *HOSO*, high-oleic sunflower oil; *AB*, allanblackia oil; *SO*, sunflower oil; *SAFA*, saturated fatty acids; *MUFA*, monounsaturated fatty acids; *PUFA*, polyunsaturated fatty acids; *SMP*, slip melting point.

Supplemental table 3 Characteristics at time of screening of participants that completed the study[a]

	All participants (n=32)	Men (n=19)	Women (n=13)
Age (y)	62 ± 5.5	61 ± 5.9	63 ± 4.9
BMI (kg/m^2)	25.6 ± 2.4	25.6 ± 1.8	25.7 ± 3.2
TC (mmol/L)	5.64 ± 1.04	5.55 ± 1.12	5.78 ± 0.96
HDL-C (mmol/L)	1.66 ± 0.39	1.53 ± 0.35	1.84 ± 0.40
Ratio TC/HDL-C	3.6 ± 1.1	3.8 ± 1.1	3.3 ± 1.0
TAG (mmol/L)	1.14 ± 0.54	1.28 ± 0.63	0.95 ± 0.27
Hb1Ac (mmol/mol)	37.5 ± 3.9	37.4 ± 4.6	37.7 ± 2.7

[a]Values are means ± SD.

BMI, body mass index; *TC*, total cholesterol; *HDL-C*, high-density lipoprotein cholesterol; *TAG*, triacylglycerol; *Hb1Ac*, glycated hemoglobin.

Supplemental table 4 Postprandial responses (iAUCs) and maximal increases of triacylglycerols (TAG) after two consecutive, identical meals rich in palmitic acid or stearic acid, provided at baseline (0 minutes) and 240 minutes after the first meal

**TAG**[a]	**Palmitic acid**	**Stearic acid**	**Difference C18:0-C16:0**[b]	
	**LSM**<br>**95% CI**	**LSM**<br>**95% CI**	**LSM**<br>**95% CI**	**P-value**
**$iAUC_{0\text{-}8h}$**<br>(mmol/(L*480 min))	349<br>289 to 409	263<br>203 to 323	-86<br>-137 to -35	0.002
**$iAUC_{0\text{-}4h}$**<br>(mmol/(L*240 min))	68<br>54 to 83	49<br>35 to 63	-19<br>-33 to -6	0.007
**$iAUC_{4\text{-}8h}$**<br>(mmol/(L*240 min))	107<br>80 to 134	86<br>59 to 113	-20<br>-47 to 6	0.127
**$TAG_{max0\text{-}8h}$**<br>(mmol/L)	1.77<br>1.50 to 2.04	1.41<br>1.15 to 1.68	-0.36<br>-0.58 to -0.13	0.003
**$TAG_{max0\text{-}4h}$**<br>(mmol/L)	0.80<br>0.66 to 0.94	0.60<br>0.46 to 0.74	-0.20<br>-0.35 to -0.06	0.007
**$TAG_{max4\text{-}8h}$**<br>(mmol/L)	0.97<br>0.80 to 1.14	0.82<br>0.65 to 0.99	-0.15<br>-0.32 to 0.02	0.079

[a]Values are shown as least squared mean (LSM) with 95% confidence interval (CI).

[b]Differences in iAUCs were tested using linear mixed models. N=32.

$iAUC_{0\text{-}8h}$ incremental AUC during the postprandial follow-up (0-8 hrs); $iAUC_{0\text{-}4h}$ incremental AUC after the 1st meal (0-4 hrs); $iAUC_{4\text{-}8h}$ incremental AUC after the 2nd meal (4-8 hrs); $TAG_{max0\text{-}8h}$ maximal increase during the postprandial follow-up (0-8 hrs); $TAG_{max0\text{-}4}$ maximal increase after the 1st meal (0-4 hrs); $TAG_{max4\text{-}8}$ maximal increase after the 2nd meal (4-8 hrs).

Supplemental table 5 Postprandial responses (iAUCs) and maximal increases of apolipoprotein B48 (apoB48) after two consecutive, identical meals rich in palmitic acid or stearic acid, provided at baseline (0 minutes) and 240 minutes after the first meal

**ApoB48**[a]	**Palmitic acid**	**Stearic acid**	**Difference C18:0-C16:0**[b]	
	**LSM**<br>**95% CI**	**LSM**<br>**95% CI**	**LSM**<br>**95% CI**	**P-value**
**$iAUC_{0-8h}$ (mg/(L*480 min))**	1472<br>1278 to 1666	1187<br>993 to 1381	-285<br>-489 to -81	0.008
**$iAUC_{0-4h}$ (mg/(L*240 min))**	390<br>332 to 448	310<br>252 to 367	-81<br>-141 to -21	0.010
**$iAUC_{4-8h}$ (mg/(L*240 min))**	512<br>408 to 616	452<br>347 to 556	-61<br>-193 to 71	0.355
**$ApoB48_{max0-8h}$** (mg/L)	5.96<br>5.15 to 6.78	5.12<br>4.30 to 5.93	-0.85<br>-1.63 to -0.69	0.034
**$ApoB48_{max0-4h}$** (mg/L)	3.03<br>2.57 to 3.48	2.47<br>2.01 to 2.93	-0.56<br>-1.11 to -0.05	0.048
**$ApoB48_{max4-8h}$** (mg/L)	3.53<br>2.84 to 4.23	3.29<br>2.60 to 3.99	-0.24<br>-1.14 to 0.66	0.585

[a]Values are shown as least squared mean (LSM) with 95% confidence interval (CI).

[b]Differences in iAUCs were tested using linear mixed models. N=32.

$iAUC_{0-8h}$ incremental AUC during the postprandial follow-up (0-8 hrs); $iAUC_{0-4h}$ incremental AUC after the 1st meal (0-4 hrs); $iAUC_{4-8h}$ incremental AUC after the 2nd meal (4-8 hrs); $ApoB48_{max0-8h}$ maximal increase during the postprandial follow-up (0-8 hrs); $ApoB48_{max0-4}$ maximal increase after the 1st meal (0-4 hrs); $ApoB48_{max4-8}$ maximal increase after the 2nd meal (4-8 hrs).

Supplemental table 6 Postprandial responses (iAUCs) and maximal increases of glucose after two consecutive, identical meals rich in palmitic acid or stearic acid, provided at baseline (0 minutes) and 240 minutes after the first meal

Glucose[a]	Palmitic acid	Stearic acid	Difference C18:0-C16:0[b]	
	LSM<br>95% CI	LSM<br>95% CI	LSM<br>95% CI	P-value
**$iAUC_{0\text{-}8h}$**<br>(mmol/(L*480 min))	220<br>170 to 271	202<br>152 to 253	-18<br>-60 to 23	0.375
**$iAUC_{0\text{-}4h}$**<br>(mmol/(L*240 min))	68.7<br>50.2 to 87.2	76.3<br>57.8 to 94.8	7.6<br>-9.2 to 24.5	0.362
**$iAUC_{4\text{-}8h}$**<br>(mmol/(L*240 min))	232<br>183 to 282	200<br>150 to 249	-33<br>-72 to 6	0.095
**$Gluc_{max0\text{-}8h}$**<br>(mmol/L)	2.43<br>2.10 to 2.76	2.46<br>2.12 to 2.79	0.03<br>-0.32 to 0.37	0.876
**$Gluc_{max0\text{-}4h}$**<br>(mmol/L)	1.90<br>1.63 to 2.18	2.19<br>1.92 to 2.46	0.29<br>-0.01 to 0.59	0.059
**$Gluc_{max4\text{-}8h}$**<br>(mmol/L)	2.50<br>2.07 to 2.93	2.11<br>1.68 to 2.54	-0.39<br>-0.80 to 0.02	0.064

[a]Values are shown as least squared mean (LSM) with 95% confidence interval (CI).

[b]Differences in iAUCs were tested using linear mixed models. N=32.

$iAUC_{0\text{-}8h}$ incremental AUC during the postprandial follow-up (0-8 hrs); $iAUC_{0\text{-}4h}$ incremental AUC after the 1st meal (0-4 hrs); $iAUC_{4\text{-}8h}$ incremental AUC after the 2nd meal (4-8 hrs); $Gluc_{max0\text{-}8h}$ maximal increase during the postprandial follow-up (0-8 hrs); $Gluc_{max0\text{-}4}$ maximal increase after the 1st meal (0-4 hrs); $Gluc_{max4\text{-}8}$ maximal increase after the 2nd meal (4-8 hrs).

Supplemental table 7 Postprandial responses (iAUCs) and maximal increases of insulin after two consecutive, identical meals rich in palmitic acid or stearic acid, provided at baseline (0 minutes) and 240 minutes after the first meal

**Insulin**[a]	**Palmitic acid**	**Stearic acid**	**Difference C18:0-C16:0**[b]	
	**LSM**<br>**95% CI**	**LSM**<br>**95% CI**	**LSM**<br>**95% CI**	**P-value**
**$iAUC_{0\text{-}8h}$**<br>**(µU/(mL*480 min))**	7977<br>6300 to 9654	7622<br>5945 to 9298	-356<br>-1213 to 502	0.404
**$iAUC_{0\text{-}4h}$**<br>**(µU/(mL*240 min))**	4423<br>3401 to 5446	4636<br>3613 to 5659	212<br>-280 to 705	0.385
**$iAUC_{4\text{-}8h}$**<br>(µU/(mL*240 min))	4085<br>3296 to 4874	3417<br>2628 to 4206	-668<br>-1376 to 41	0.064
**$Ins_{max0\text{-}8h}$**<br>(µU/mL)	79.9<br>62.9 to 96.9	84.2<br>67.3 to 101	4.4<br>-8.2 to 16.9	0.483
**$Ins_{max0\text{-}4h}$**<br>(µU/mL)	79.1<br>62.1 to 96.2	84.2<br>67.2 to 101	5.1<br>-7.7 to 18.0	0.422
**$Ins_{max4\text{-}8h}$**<br>(µU/mL)	43.9<br>33.9 to 53.8	36.4<br>26.5 to 46.4	-7.4<br>-16.8 to 1.9	0.115

[a]Values are shown as least squared mean (LSM) with 95% confidence interval (CI).

[b]Differences in iAUCs were tested using linear mixed models. N=32.

$iAUC_{0\text{-}8h}$ incremental AUC during the postprandial follow-up (0-8 hrs); *$iAUC_{0\text{-}4h}$* incremental AUC after the 1st meal (0-4 hrs); *$iAUC_{4\text{-}8h}$* incremental AUC after the 2nd meal (4-8 hrs); *$Ins_{max0\text{-}8h}$* maximal increase during the postprandial follow-up (0-8 hrs); *$Ins_{max0\text{-}4}$* maximal increase after the 1st meal (0-4 hrs); *$Ins_{max4\text{-}8}$* maximal increase after the 2nd meal (4-8 hrs).

Supplemental table 8 Postprandial responses (iAUCs) and maximal increases of C-peptide after two consecutive, identical meals rich in palmitic acid or stearic acid, provided at baseline (0 minutes) and 240 minutes after the first meal

**C-peptide**[a]	**Palmitic acid**	**Stearic acid**	**Difference C18:0-C16:0**[b]	
	**LSM**<br>**95% CI**	**LSM**<br>**95% CI**	**LSM**<br>**95% CI**	**P-value**
**$iAUC_{0\text{-}8h}$**<br>(mg/(mL*480 min))	970<br>845 to 1095	959<br>834 to 1084	-11<br>-114 to 92	0.825
**$iAUC_{0\text{-}4h}$**<br>(mg/(mL*240 min))	452<br>385 to 520	471<br>404 to 539	19<br>-24 to 62	0.379
**$iAUC_{4\text{-}8h}$**<br>(mg/(mL*240 min))	582<br>520 to 644	537<br>475 to 599	-45<br>-109 to 19	0.162
**$C\text{-}pep_{max0\text{-}8h}$**<br>(ng/mL)	6.07<br>5.22 to 6.92	6.20<br>5.36 to 7.05	0.13<br>-0.61 to 0.87	0.717
**$C\text{-}pep_{max0\text{-}4h}$**<br>(ng/mL)	5.84<br>4.99 to 6.69	6.11<br>5.26 to 6.96	0.27<br>-0.49 to 1.02	0.472
**$C\text{-}pep_{max4\text{-}8h}$**<br>(ng/mL)	5.02<br>4.43 to 5.62	4.66<br>4.07 to 5.25	-0.36<br>-0.94 to 0.22	0.213

[a]Values are shown as least squared mean (LSM) with 95% confidence interval (CI).

[b]Differences in iAUCs were tested using linear mixed models. N=32.

$iAUC_{0\text{-}8h}$ incremental AUC during the postprandial follow-up (0-8 hrs); $iAUC_{0\text{-}4h}$ incremental AUC after the 1st meal (0-4 hrs); $iAUC_{4\text{-}8h}$ incremental AUC after the 2nd meal (4-8 hrs); $C\text{-}pep_{max0\text{-}8h}$ maximal increase during the postprandial follow-up (0-8 hrs); $C\text{-}pep_{max0\text{-}4}$ maximal increase after the 1st meal (0-4 hrs); $C\text{-}pep_{max4\text{-}8}$ maximal increase after the 2nd meal (4-8 hrs).

Supplemental table 9 Postprandial responses (dAUCs) and maximal decreases of non-esterified fatty acids (NEFA) after two consecutive, identical meals rich in palmitic acid or stearic acid, provided at baseline (0 minutes) and 240 minutes after the first meal

NEFA	Palmitic acid	Stearic acid	Difference C18:0-C16:0	
	**LSM**<br>**95% CI**	**LSM**<br>**95% CI**	**LSM**<br>**95% CI**	**P-value**
**$dAUC_{0\text{-}8h}$**<br>(mmol/(L*480 min))	46.5<br>27.7 to 65.4	83.4<br>64.5 to 102	36.9<br>12.3 to 61.4	0.005
**$dAUC_{0\text{-}4h}$**<br>(mmol/(L*240 min))	31.0<br>21.6 to 40.3	45.2<br>35.9 to 54.6	14.3<br>1.9 to 26.6	0.025
**$dAUC_{4\text{-}8h}$**<br>(mmol/(L*240 min))	23.2<br>14.7 to 31.6	31.0<br>22.5 to 39.4	7.8<br>2.5 to 13.1	0.005
**$NEFA_{max0\text{-}8h}$**<br>(μmol/L)	-268<br>-318 to -217	-340<br>-391 to -290	-73<br>-134 to -10	0.026
**$NEFA_{max0\text{-}4h}$**<br>(μmol/L)	-263<br>-313 to -214	-329<br>-378 to -279	-65<br>-131 to 1	0.054
**$NEFA_{max4\text{-}8h}$** (μmol/L)	-232<br>-292 to -172	-246<br>-306 to -186	-14<br>-55 to 28	0.499

[a]Values are shown as least squared mean (LSM) with 95% confidence interval (CI).

[b]Differences in dAUCs were tested using linear mixed models. N=32.

$dAUC_{0\text{-}8h}$ decremental AUC during the postprandial follow-up (0-8 hrs); *$dAUC_{0\text{-}4h}$* decremental AUC after the 1st meal (0-4 hrs); *$dAUC_{4\text{-}8h}$* decremental AUC after the 2nd meal (4-8 hrs); *$NEFA_{max0\text{-}8h}$* maximal decrease during the postprandial follow-up (0-8 hrs); *$NEFA_{max0\text{-}4}$* maximal decrease after the 1st meal (0-4 hrs); *$NEFA_{max4\text{-}8}$* maximal decrease after the 2nd meal (4-8 hrs).

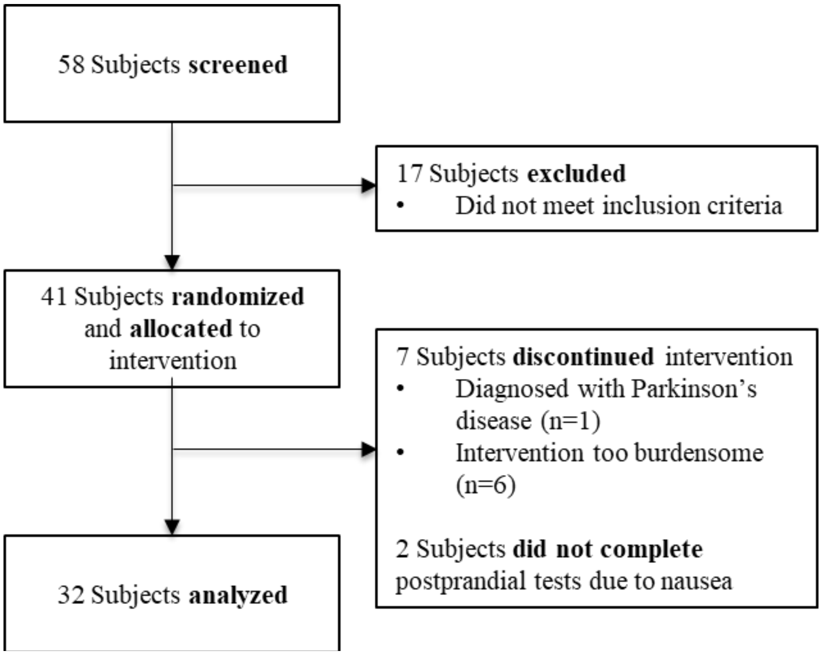

Supplemental figure 1 Flow chart of subjects throughout the study

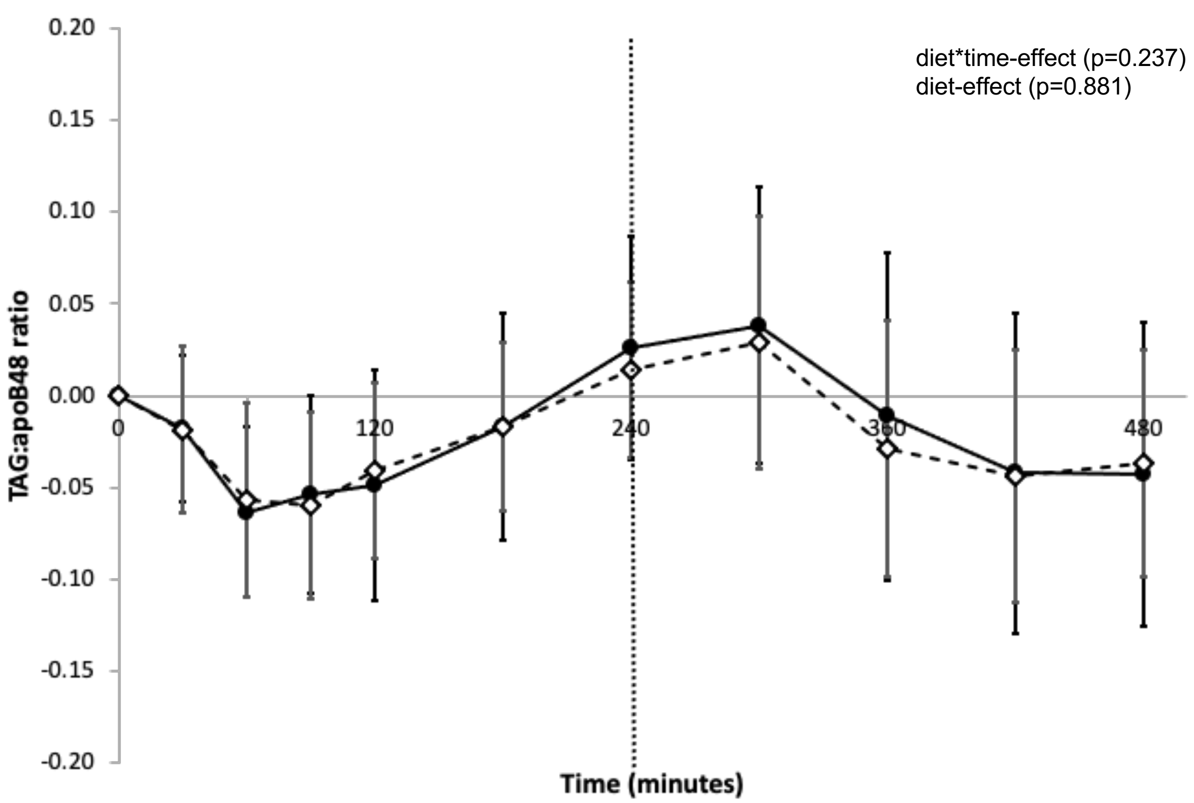

Supplemental figure 2. Postprandial changes in the ratio triacylglycerol:apolipoprotein B48 (TAG:apoB48 ratio) over time after meals rich in palmitic acid (•) or stearic acid (◊)[a]

[a]TAG and apoB48 concentrations were measured at baseline, and 30, 60, 90, 120, 180, 240, 300, 360, and 480 minutes after meal intake. After 240 minutes, a second meal was consumed that was similar to the first meal. Postprandial time curves were analyzed using linear mixed models. N=32. A significant overall time-effect was observed ($p<0.001$), e.g. TAG:apoB48 ratio was 0.094 points higher (95% CI 0.069, 0.119; $p<0.001$) 1 hour after the second meal (T=300) compared to 1 hour after the first meal (T=60).
